# Intracranial varicella zoster virus infection may elicit an unusual hyperkinetic volitional tremor

**DOI:** 10.1016/j.prdoa.2020.100069

**Published:** 2020-08-12

**Authors:** Ryogo Shobatake, Hiroshi Kataoka, Tesseki Izumi, Eura Nobuyuki, Makoto Kawahara, Kazuma Sugie

**Affiliations:** a Department of Neurology, Nara Medical University, Nara Medical University, Kashihara, Nara, Japan; b Department of Neurology, Nara City Hospital, Nara, Nara, Japan

**Keywords:** Varicella zoster virus, Tremor, Encephalitis, Hyperkinésie volitionnelle, Involuntary movement

## Abstract

Central nervous system manifestations of varicella zoster virus (VZV) infection are uncommon, and associated involuntary movement is rare. Herein, we describe a patient with VZV induced encephalopathy who presented with an unusual hyperkinetic volitional tremor.

## 1 Case

A 61-year-old woman with a history of hypertension presented with a painful herpes zoster rash on her hip. Four days later, she received oral valaciclovir and topical vidarabine. In the evening, she became drowsy, and involuntary movement in her right upper limb was evident, leading to admission to our hospital. Her body temperature and blood pressure increased to 37.6 °C and 151/84 mmHg, respectively. Her level of consciousness was senselessness, but she could follow our instructions. Neck stiffness was absent. Slurred and scanning speech and ataxic gait were present. Superficial and vibratory sensations were not impaired. A hyperkinetic tremor was observed in her right limb. It occurred and developed when intentionally approaching an object (supplemental video figure). Serum laboratory findings showed an increase in C-reactive protein (1.9 mg/dl). A cerebrospinal fluid (CSF) analysis at admission showed five lymphocytes and a protein concentration of 62 mg/dl. Cranial and cervical enhanced magnetic resonance imaging showed neither abnormal intensity nor enhancement. Serum and CSF VZV-specific IgG antibodies for were >128 and 0.35, respectively, and VZV-specific IgM antibody was not evident in both samples. An electroencephalogram showed no epileptic forms. A surface electromyogram showed a tremor-type waveform with reciprocity and rhythm of the extensor and flexor muscles at 6 Hz. Three days after the initiation of intravenous acyclovir (10 mg/kg/day three times), her consciousness and speech impairments improved. Two weeks after admission, a second CSF examination revealed that the level of lymphocytes (14/μL) and VZV-specific IgG antibody (7.86) were increased, and real-time polymerase chain reaction test was positive in the CSF (660,000 copies/ml). The involuntary movement in her right upper limb persisted despite continuation of acyclovir. Because of the elevated interleukin 6 level (133 pg/ml) in the first CSF analysis, intravenous steroid pulse treatment (two courses of 1 g/day for 3 days) was added. Thereafter, the severity of the involuntary movement markedly reduced, and CSF examination showed that the level of lymphocytes and VZV-specific IgG antibody for VZV were decreased to 7/μL and 1.29, respectively, and the interleukin 6 level decreased to 0.856 pg/ml. Anti-*N*-methyl-d-aspartate receptor (anti-NMDAR) antibody was not detected in CSF. After acyclovir treatment, the severity of the skin rash largely decreased, but oral pregabalin was required for persistent skin pain during the disease course. In January 2020, she was free of symptoms including the involuntary movement.

## 2 Discussion

The hand tremor became more prominent while intentionally approaching an object, which was previously referred to as hyperkinésie volitionnelle [1]. The responsible lesion was considered to be in the cerebellar efferent system (dentate nucleus-red nucleus-thalamic tract), and the observed slurred and scanning speech and ataxic gait without peripheral vibratory impairment supports this indication. A neuroimaging study showed a relationship between tremor and cerebellar activation in patients with an essential tremor [2]. Pathologically degenerative changes such as loss or axonal torpedoes of Purkinje cells were observed in patients with an essential tremor [3]. Patients with cerebellar intension tremor had lesions in the fiber tracts from the deep cerebellar nuclei, including the dentate nucleus, to contralateral ventrolateral thalamus [3]. A signal change and structural alternation on MRI could not be detected, but the cerebellum receiving inputs from many sources projects to the thalamus and red nucleus, and these support that the present hyperkinetic tremor could be associated with the dentate nucleus-red nucleus-thalamic tract.

Myorhythmia, which is defined as repetitive, rhythmic, and jerky movements, is observed in patients with encephalitis [4], but it was unlikely in the present case because it occurs at rest and is characterized by a slow frequency (1 to 4 Hz). The observation of the large change in the IgG antibody titer during the disease course, that is, 21-fold increase and subsequent 6-fold decrease, means that the VZV infection might be directly involved with the responsible lesion of the hyperkinetic tremor. However, it is not likely because the serum and CSF IgM antibody was not evident. The hyperkinetic volitional tremor responded to steroid treatment with a decreasing titer of CSF interleukin 6. Whether this case involved VZV or a secondary intrathecal immune reaction directly related to the tremor is quite uncertain. In addition, encephalitis with neuronal antibodies can show a tremor [5], and anti-NMDAR encephalitis, which was not evident of this antibody in the present case, reportedly can concomitantly occur with VZV infection [6]. VZV induced encephalopathy can present with an unusual lateral tremor, and it potentially improves with additional immunotherapy.

The following is the supplementary data related to this article.

Video 1 This hand tremor did not occur at rest and became more prominent as the fingertip intentionally approached the object. After treatment, the severity of this hyperkinetic volitional tremor markedly decreased. Video 1

## Author responsibilities and contributions

R Shobatake and H Kataoka were responsible for the overall study design, and wrote the manuscript.

R Shobatake. T Izumi, N Eura, M Kawahara and H Kataoka contributed to running the study and acquisition of data.

R Shobatake and H Kataoka contributed to analysis and interpretation of data.

H Kataoka, and K Sugie contributed to drafting and critical revision of part of the submitted materials.

## Ethics statement

No investigations or interventions were performed outside of routine clinical care for this patient. As this is a case report, without experimental intervention into routine care, no formal research ethics approval was required. Fully informed consent was received from the patient. This case study reports routine clinical care provided for a patient only.

## Financial disclosures

There was no financial disclosures related with our manuscript.

## Declaration of competing interest

The authors report no conflicts of interest.
